# Restricted Diffusion in the Splenium of the Corpus Callosum After Cardiac Arrest 

**DOI:** 10.2174/1874440000802010001

**Published:** 2008-01-11

**Authors:** Matt T Bianchi, John R Sims

**Affiliations:** 1 Department of Neurology, Massachusetts General Hospital and Brigham and Women’s Hospital, Fruit Street, Wang Ambulatory Center 8th Floor, Boston, MA 02114, USA; 2 Departments of Neurology and Radiology, Massachusetts General Hospital, CNY49 Rm6403, Charlestown, MA 02129, USA

## Abstract

The value of MRI findings for coma prognostication is a question of great clinical and pathological relevance. We describe MRI evidence of restricted diffusion in the splenium in 5 patients with coma after cardiopulmonary resuscitation following cardiac arrest. The most common clinical presentation of corpus callosum lesions (of any cause) is altered mental status, consistent with the global importance of these extensive inter-hemispheric fibers. In our four cases with bilateral splenium restricted diffusion, none of the patients recovered consciousness. One patient with a unilateral (likely embolic) restricted diffusion lesion had excellent recovery. In contrast to unilateral ischemic callosal lesions, we believe that generalized, midline splenium restricted diffusion occurring after cardiopulmonary arrest represents Wallerian degeneration of interhemispheric neurons rather than direct ischemic damage to the white matter or axons of the callosum and thus will likely portend a poor prognosis.

## INTRODUCTION

Despite increasing availability of MRI technology, the prognostic value of brain imaging in coma after cardiac arrest remains uncertain. Global hypoxic-ischemic damage is a potential consequence of cardiac arrest. The corpus callosum is not highly susceptible to such injury, and indeed ischemic lesions of the callosum are uncommon and midline ischemic lesions are rare [1-3]. In contrast, MRI lesions of the corpus callosum have been reported in situations of trauma, inflammation, malignancy, metabolic disorders, infections and toxic exposure [3-6]. The brain regions thought to be most sensitive to hypoxic/ischemic injury are the CA1 and CA3 regions of the hippocampus, cortical layers 3 and 5, medium spiny neurons of the striatum, and the Purkinje layer of the cerebellum. The corpus callosum, particularly the midline, appears to be relatively resistant to ischemic injury [7, 8]. Although ischemia to the corpus callosum has been reported in up to 9% of ischemic strokes, the majority of these unilateral lesions occurred in conjunction with posterior cerebral artery (PCA) territory infarcts [2]. The pericallosal or the subcallosal and medial callosal, branches of the anterior cerebral artery (ACA) and anterior communicating artery, respectively, supply the anterior two thirds of the callosum. The posterior pericallosal artery, a branch of the PCA, supplies the posterior callosum, including the splenium [9]. Furthermore, there is a significant pericallosal anastamotic plexus between these main blood supplies.

The most common clinical presentation of corpus callosum lesions (of any cause) was altered mental status [10], consistent with the global importance of these extensive interhemispheric fiber tracks. Therefore imaging evidence of injury to this region might prove clinically useful for predicting neurological deficits in some settings. Here we describe the clinical and radiological findings in five patients with splenium injury in the setting of resuscitation from cardiac arrest and discuss the clinco-pathological consequences of such lesions.

### Case 1: Persistent Coma

**Fig. (1):**
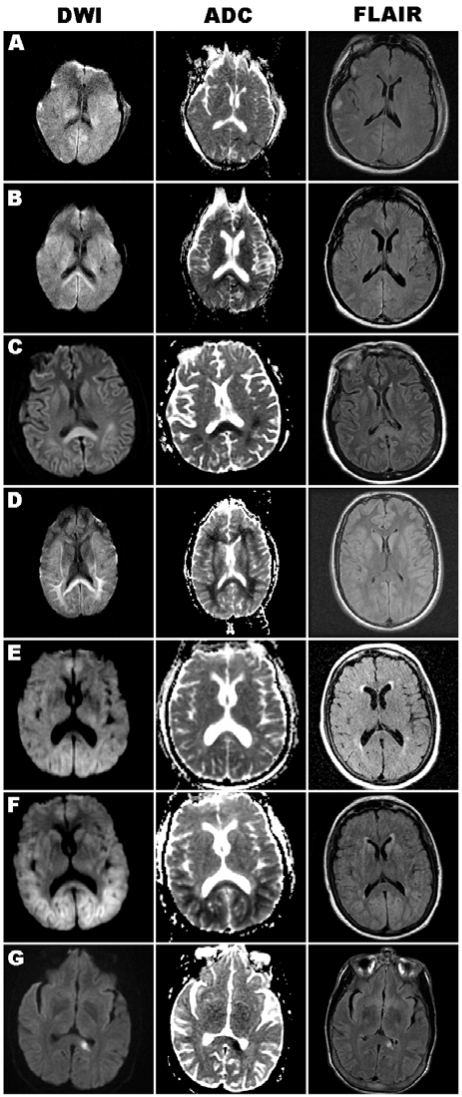
Axial brain MRI images of DWI, ADC, and FLAIR sequences obtained in each case. A, Case 1, early MRI (Day 3) without splenium or cortical diffusion signal. B, Repeat MRI of case 1 (Day 10), with bilateral DWI-bright and ADC-dark splenium signal. C, Case 2 (Day 7), prominent DWI-bright and ADC-dark splenium signal, with subtle cortical ribbon signal, hyperintensity of these regions also present on FLAIR sequence. D, Case 3 (Day 8), DWI-bright and ADC-dark signal in the splenium and subcortical white matter, abnormal signal not well seen on FLAIR. E, Case 4, early MRI (Day 1) with minimal pathology. F, Case 4, late MRI (Day 6) with DWI-bright and ADC-dark signal in the splenium and occipital/parietal regions, with subtle corresponding hyperintensity signal on FLAIR. G, Case 5 (Day 3), isolated ischemic lesion in the left splenium, seen on all three sequences.

A previously healthy 71-year-old man without cardiovascular risk factors collapsed while jogging and received CPR immediately by witnesses. Paramedics provided cardioversion for ventricular fibrillation (VF) arrest. The echocardiogram suggested inferior ischemia, which was managed medically. Initial examination revealed intact brainstem reflexes, partially open eyes, and no motor response to pain (Glasgow Coma Scale (GCS)=6). EEG on hospital day 3 showed low amplitude and diffuse, anterior predominant alpha frequency, without epileptiform activity. MRI performed on hospital day 3 showed no splenium lesion (Fig. **1A**). The patient’s examination was unchanged over the following week. Repeated EEGs showed low voltage slowing R>L with questionable intermittent reactivity, but without epileptiform activity. Failure to clinically improve prompted a repeat MRI on hospital day 10, which was notable for restricted diffusion in the bilateral splenium (Fig. **1B**). Three days later, the patient expired after care was directed toward comfort measures only.

### Case 2: Persistent Coma

A 61-year-old female smoker with hypertension and hyperlipidemia was resuscitated after ventricular tachycardia (VT)/VF arrest complicated by persistent hypotension. Resuscitation was rapidly initiated but prolonged (45 minutes) and required 14 electrical cardioversions. Cardiac catheterization revealed LAD occlusion that was stented, and she required repeated peri-procedure cardioversion and intra-aortic balloon pump and vasopressor support. Initial examination revealed absent cranial nerve reflexes and flexor posturing to pain (GCS=5T). The next day, cranial nerves were intact; eyes opened spontaneously but did not track, and she had flexor posturing to pain (GCS=8). Initial head CT performed 48 hours after arrest was normal. EEG revealed diffuse slowing without epileptiform activity. MRI performed on hospital day 7 revealed prominent restricted diffusion in the bilateral splenium, with mild diffuse restriction in the bilateral cortical ribbon, basal ganglia, and thalami (Fig. **1C**). The neurological examination did not improve, and the patient expired on day 15 after care was directed toward comfort measures only.

### Case 3: Persistent Coma

A 46-year-old man with hypertension was resuscitated after VT/VF arrest and underwent emergent coronary catheter revascularization. Initial examination revealed intact brainstem reflexes and withdrawal from pain (GCS=6T). By the 5^th^ day, he developed extensor posturing in the arms and triple flexion in the legs to noxious stimuli. There was no cardiac or respiratory compromise during the intervening observation time in the intensive care unit to explain his deterioration. Invasive monitoring ruled out raised intracranial pressure. MRI performed on hospital day 8 revealed dramatic restricted diffusion in the bilateral, particularly posterior, white matter tracks, including prominent signal in the bilateral splenium (Fig. **1D**). SSEPs were normal on day 11. The examination did not improve. He expired after care was directed toward comfort measures only.

### Case 4: Persistent Coma

A 69-year-old woman with remote carotid endarterectomy was admitted for lung biopsy related to idiopathic progressive hypoxic respiratory dysfunction. One day after the uncomplicated procedure, she was resuscitated from asystolic cardiac arrest. Initial examination revealed eyes open to noxious stimulation, extensor posturing, and intact brainstem reflexes (GCS=4T). MRI performed 24 hours post-arrest showed no splenium lesion (Fig. **1E**). By the 4th day post-arrest, she showed slight improvement by withdrawing to pain, but had developed spontaneous downward gaze. MRI was performed six days after arrest, and revealed diffuse restricted diffusion in the cortical ribbon and subcortical nuclei, as well as the bilateral splenium (Fig. **1F**). By day 7 she had deteriorated, showing flexor posturing, without any evidence of cardiac or respiratory compromise to explain the clinical decline. Examination did not improve and she expired after care was directed toward comfort measures only.

### Case 5: Rapid Recovery

A 27-year-old man with Alport’s disease, on hemodialysis, was resuscitated following VT/VF and pulseless electrical activity (PEA) arrest in the setting of extreme hyperkalemia. His resuscitation spanned over 90 minutes, with recurrent periods of PEA arrest. Initial examination revealed eyes opening to noxious stimuli, extensor posturing, and intact brainstem reflexes (GCS=4T). Head CT revealed global cerebral edema, and mannitol was administered empirically. Repeat head CT 20 hours after the first scan showed resolution of edema, and the patient was moving purposefully and following commands. After extubation the next day, he was conversant but exhibited significant short-term memory loss. MRI performed 1 day post-arrest revealed a small region of restricted diffusion in the left splenium (Fig. **1G**), without any other T2 or DWI abnormalities. His main neurological deficit was short-term memory loss.

## DISCUSSION

Although the imaging characteristics of hyperintensity on diffusion weighted images and hypointensity on ADC maps is suggestive of ischemia, the actual mechanism of the splenium lesions is uncertain in the four case fatalities. Several features argue against ischemia [2] as the lesion etiology in these fatalities: 1) symmetric midline-spanning lesions do not reflect a single vascular territory; 2) lack of involvement of neighboring vascular territories; 3) the delayed appearance of the splenium lesions in two of the cases in which both early and late MRIs were available (also likely in case 2 given normal CT 48 hours after arrest); 4) the relative resistance of the corpus callosum to hypoxic-ischemic injury (relative to cortex, hippocampus, striatum and cerebellar Purkinje cells). None of the fatalities showed significant posterior circulation stenoses on vessel imaging. The small unilateral splenium lesion (case 5) was suggestive of an embolic event, and stands in mechanistic and prognostic contrast to the prior 4 cases despite the lesion occurring in the setting of resuscitation.

Although pathological data was not available in these cases, we hypothesize that the splenium findings reflected early Wallerian degeneration from diffuse cortical neuronal death from global hypoxic-ischemic injury, particularly the highly susceptible transcallosal pyramidal neurons in cortical layer three. Although central nervous system Wallerian degeneration occurs most commonly on a longer time scale (weeks), early subacute changes (over days) reflecting Wallerian degeneration have been reported following brain ischemia [11, 12]. The radiological manifestations of restricted diffusion may be most detectable where degenerating processes from these widely distributed cortical neurons are most concentrated, in the corpus callosum. Pathological investigation of similar cases in the future may shed light on the mechanism(s) of the restricted diffusion. An alternative hypothesis is that the imaging changes reflect delayed cytotoxic edema. The delayed appearance of the lesions in the two cases where early and later MRIs were available argues against acute watershed ischemia at the time of the circulatory arrest. Although extra-callosal diffusion changes were present on imaging, the bilateral splenium lesions represented the one common anatomical location among the four fatalities. The case with prominent cortical restricted diffusion (case 4) showed preferential posterior (parietal/occipital) changes, which might explain the preferential imaging change in the splenium. In the other cases, it remains uncertain why global hypoxia-ischemia would be reflected in the splenium rather than the entire callosum. Although the mechanism of this finding awaits further study, similar intra-callosal differences in radiological markers of corpus callosum damage have been described by MRI techniques in the chronic degeneration of Alzheimer’s dementia [13]. In addition to pathological assessment, future studies might benefit from other imaging modalities such as MR spectroscopy (in cortex and splenium for example) or PET studies. For patients sufficiently stable for frequent radiological assessment, the temporal course of abnormalities revealed through repeated imaging may provide additional mechanistic insights to complement pathology.

A correlation between corpus callosum damage and poor neurological prognosis has been suggested in trauma [14-17] and Marchiafava-Bignami disease [18]. The latter lesions are demyelinating and necrotic with chromatolysis, similar to that seen in Wallerian degeneration and with anoxic brain injury, and these lesions can also present with restricted diffusion [19]. These studies lend support for the hypothesis that late bilateral splenium DWI lesions may be a specific predictor of poor neurological outcome in coma after cardiac arrest.

Recovery from coma after cardiac arrest may involve many factors related to the patient’s premorbid condition and the circumstances of the arrest and resuscitation. Of note, none of the patients underwent cooling protocol. Although none of the cases met early (day 1-3) criteria for poor prognosis according to the AAN guidelines [20] for coma prognostication after cardiac arrest, cases 1 and 3 eventually met criteria for poor prognosis on days 3-5 post-arrest by virtue of extensor posturing to pain. Prognostication of neurological function after brain injury remains an important clinical challenge. The American Academy of Neurology recently released its first guideline for neurological prognostication for coma after resuscitation from cardiac arrest [20]. Among the numerous variables reviewed in this evidence-based guideline, MRI studies had limited supportive literature, and thus MRI findings are not considered in the algorithm. Despite this lack of evidence, the high sensitivity of MRI sequences for various forms of brain injury may provide promising information in ongoing studies of radiological indicators of prognosis.
